# Pre-Vaccination Stress, Post-Vaccination Adverse Reactions, and Attitudes towards Vaccination after Receiving the COVID-19 Vaccine among Health Care Workers

**DOI:** 10.3390/vaccines10030401

**Published:** 2022-03-06

**Authors:** Sylwia Kałucka, Ewa Kusideł, Agnieszka Głowacka, Paulina Oczoś, Izabela Grzegorczyk-Karolak

**Affiliations:** 1 Department of Coordinated Care, Medical University of Lodz, 90-251 Lodz, Poland; 2 Department of Spatial Econometrics, Faculty of Economics and Sociology, University of Lodz, 90-255 Lodz, Poland; ewa.kusidel@eksoc.uni.lodz.pl; 3 Department of Developmental Nursing and Health Promotion, Medical University of Lodz, 90-251 Lodz, Poland; agnieszka.glowacka@umed.lodz.pl; 4 Student’s’ Scientific Society at Department of Coordinated Care Medical, University of Lodz, 90-251 Lodz, Poland; paulina.oczos@stud.umed.lodz.pl; 5 Department of Biology and Pharmaceutical Botany, Medical University of Lodz, 90-151 Lodz, Poland; izabela.grzegorczyk@umed.lodz.pl

**Keywords:** COVID-19 vaccine, attitude, stress, vaccination adverse reaction, BioNTech/Pfizer vaccine, Oxford/AstraZeneca vaccine, doctor, nurse, midwife, medical students, nursing and midwifery students, healthcare workers

## Abstract

The vaccines against COVID-19 are the best treatment for limiting the spread of the epidemic, and from an individual point of view, for avoiding getting sick. A cross-sectional retrospective survey was conducted from 15 May to 15 July 2021 among healthcare workers, including doctors, nurses, midwives, and students at the Medical University of Lodz (nursing, midwifery and medical students) in Poland. Data were obtained from 1080 participants. The aim of the study was to evaluate vaccination coverage against COVID-19 among healthcare workers (HCWs) in Poland, and to analyze their attitude towards the available vaccines, stress before taking, and side effects after administrating them, and motivation towards continuing vaccination in the future (if necessary). The survey also estimated the frequency and quality of adverse post-vaccination reactions after two doses of BioNTech/Pfizer and two doses of AstraZeneca vaccines. The present study revealed that the vaccination ratio after 6 months from the start of vaccination against COVID-19 in Poland among HCWs was very high at 91.2%. However, doctors and medical students were more likely to be vaccinated than nurses and midwives, and nursing and midwifery students (94.8%, 98.3% vs. 78.9% and 86.3%, respectively). The main reasons that HCWs reported receiving a vaccination were to protect the health of themselves and their families, while the main reasons for avoiding a vaccination were fear of side effects, doubts about effectiveness, and an expedited clinical trial process of vaccines. Furthermore, more than two-thirds of those vaccinated reported side effects after receiving at least one dose of the vaccine. Most of the side effects were short-term symptoms with only slight and moderate intensification. The univariate and multivariate logistic regressions showed that the type of vaccine used had a significant impact on the occurrence of adverse post-vaccination effects and the severity and duration of vaccination symptoms. In addition, chronic disease and fear of vaccination also had some influence. Despite this, most participants (more often older than younger participants; *p* < 0.001) were in favor of compulsory vaccination against COVID-19 for HCWs.

## 1. Introduction

In November of 2019, the first cases of pneumonia of an unknown etiology were diagnosed in China [1]. The rapid increase in the number of human infections and deaths in Wuhan, in the central Hubei province of the country, resulted in an intensive search for the etiological factor and led to the identification of a new coronavirus, initially named 2019-nCoV [2]. This was the second identified severe acute respiratory syndrome (SARS) coronavirus, designated by the WHO as a variant of concern (VoC), and thus giving rise to the name of the new virus—the world-famous SARS-CoV-2 [3,4]. The virus is transmitted by airborne droplets, which causes acute respiratory infection coronavirus disease 2019 (COVID-19) [4,5]. In some patients, it causes severe pneumonia and acute respiratory distress syndrome, requiring mechanical ventilation. Moreover, an infection can result in further complications such as endothelial disease, wreaking indiscriminate havoc on multiple organs including the lungs, heart, brain, kidney, and vasculature [6,7].

At the time of writing, it has only been nine months since the World Health Organization announced (11 March 2020) the pandemic of SARS-CoV2, and the first vaccination against COVID-19 was administered in December 2020 [4,8,9,10]. At the present moment, vaccinations seem to be the only tool that can effectively and safely reduce the spread of the virus, with all its consequences and costs.

The vaccination program in Poland started on 27 December 2020 [11]. Until then, 1,257,799 COVID-19 cases had been reported in Poland, and 27,118 people had died. According to reports from the Ministry of Health during this period, 20,476 doctors, 50,990 nurses, and 4764 midwives had become infected, of which 66, 51, and 4 died, respectively [12]. For the first time in Poland, a national comprehensive, completely voluntary, but free vaccination program covering the whole population was introduced [11]. In this National Program, the whole population was divided into four groups according to the high level of exposure to infection and the severity course of the SARS-CoV-2 infection (including the highest risk of death). The first group, called group zero, included healthcare professionals (including medical students) who are particularly vulnerable to SARS-CoV-2 infection, and whose work is strategically important to the whole of society. The other groups were residents of nursing homes, people over 60 years of age, employees of uniformed services and teachers (group one); people under 60 years of age with chronic diseases (group two); and other adult populations (group three).

With the approval of the European Medicines Committee (EMA) and the European Commission, Poland received the SARS-CoV-2 vaccines. The Polish government first purchased the two-dose vaccines BNT162b2 mRNA COVID-19 by BioNTech/Pfizer [13], ChAdOX1nCoV-19 by AstraZeneca [14], and mRNA-1273 SARS-CoV2 vaccine by Moderna [15], followed by the single-dose Ad26.COV2.S COVID-19 vaccine by Johnson & Johnson [16]. Both BioNTech/Pfizer and Moderna vaccines contain the so-called template ribonucleic acid (mRNA) encoding the SARS-CoV-2 coronavirus coat protein. The mRNA is located in the lipid coat, enters the cell, and triggers the production of a protein in the ribosomes, which is responsible for triggering an immune system response in the vaccinated person. Meanwhile, vector vaccines such as AstraZeneca and Jonhson & Jonhson are based on the adenovirus, which triggers an immune response by transferring fragments of genetic material to human cells and serving as an information carrier for the production of SARS-CoV-2 proteins. These types of vectors had also been used in vaccines against Ebola, HIV, RSV, and Zika virus vaccines [17].

The first vaccine available in Poland was BioNTech/Pfizer, followed by Moderna and AstraZeneca. The Johnson & Johnson vaccine was used only from the second half of April 2021 [11]. However, especially at the beginning, the vaccines were delivered with a delay, in a smaller number of units than originally purchased, and supplies were frequently suspended by the producer—which all made it impossible to carry out the planned vaccinations smoothly. Individuals in group zero, including healthcare workers (HCWs), did not have a choice in the kind of vaccine they received. HCWs were initially vaccinated only with the BioNTech/Pfizer vaccine. In the second half of January, Moderna vaccines began to arrive in Poland, but in such small quantities that the probability of being vaccinated with this vaccine was very low. At the end of January, AstraZeneca vaccines were also delivered to Poland. Due to the shortage in the supply of the vaccines by BioNTech/Pfizer and Moderna, the AstraZeneca vaccines temporarily became the only available vaccine, with the BioNTech/Pfizer and Moderna vaccines only available as a second dose for those previously vaccinated. A choice of a vaccine was only possible from the turn of April and May [11].

By 15 May 2021, approximately 15.5 million vaccines against SARS-CoV-2 had been used in Poland, and about 4.4 million people were fully vaccinated [11]. At the same time, in connection with vaccination with any of the four vaccines used in Poland, 6051 adverse post-vaccination reactions were reported to the State Sanitary Inspection, of which 5161 were mild [18]. The official definition of adverse post-vaccine reactions (APVR) is a disorder health condition (often transient and mild), which occurs within four weeks after administration of the vaccine (the exception is the administration of the tuberculosis vaccine, where the time criterion is longer than four weeks) [19]. In Poland, according to Art. 21 of the Act on preventing and combating infections and infectious diseases in humans, the post-vaccination adverse reaction should be reported to Sanitary Inspection [18,20].

Cases of post-vaccination reactions in thousands of vaccinated people, along with the relatively short time in which the vaccines were created and obtained, have become a pretext for anti-vaccination movements to undermine the safety and effectiveness of COVID-19 vaccines, and a cause for inducing pre-vaccination fear in society.

Following this important epidemiological and social topic, the present study aims to assess vaccination coverage in Poland against COVID-19 among healthcare workers, nurses, doctors, midwives, and students at a medical university (nursing, midwifery, medicine). Since the availability of vaccinations, there have been numerous studies evaluating the intention to get vaccinated against SARS-CoV-2 among health professionals in different European and non-European countries; however, the results showed wide variability [21,22,23,24]. The present research is one of the few studies so far that shows the actual rate of vaccination at a time when full vaccination was possible for all HCWs who wanted to take the vaccine. Our objective was also to explore the motivations of health professionals for getting vaccinated, and their vaccine-related stress. This research estimated the frequency and intensification of adverse post-vaccination reactions associated with vaccination with BioNTech/Pfizer and AstraZeneca. In addition, this study aimed to determine the ratio of HCWs to vaccines against COVID-19 after vaccination, and their motivation to continue vaccination in the future, which may potentially translate into the attitude towards this vaccination across the whole of society.

## 2. Materials and Methods

### 2.1. Participants

The participants who took part in this study were healthcare professionals (doctors, nurses, midwives) working in the academic centers (hospitals and clinics), and students majoring in medicine, nursing, and midwifery studying in Lodz, a large academic city located in the center of Poland. The estimated total population of health professionals was 6,306. The sample size was calculated by the Raosoft sample size calculator [25]. Based on the estimated population and a response distribution of 50%, the required sample size was 363 for a confidence level of 95% and a 5% margin of error. The group of professionally active health workers was divided into two subgroups depending on the length of service: under ten years (without completion of specialization yet) and over ten years (with specialization). In turn, the group of students, who are students at the largest medical university in Poland, was divided into two subgroups: younger students (with mostly theoretical classes) and senior students (with mostly clinical classes).

### 2.2. Cross-Sectional Survey

The research was carried out from 15 May to 15 July 2021 (Supplementary Materials Figure S1). This period was chosen because almost 6 months had passed since the start of COVID-19 vaccinations for health professionals; therefore, everyone who wanted to get vaccinated, could have done so by this point. All participants in the study understood the necessity of completing the questionnaire, and understood that the return of a blank questionnaire meant they had refused to participate in the study.

### 2.3. Questionnaire Survey

An original questionnaire was prepared by the principal investigator for the purpose of this study. The questionnaire was self-administered, paper-based, and anonymous. The original questionnaire was designed based on previous studies and the opinions of four experts. One month before the main study, a pilot study that analyzed 100 randomly selected healthcare workers was conducted (Supplementary Materials Figure S1). The questionnaires were validated on the basis of standard procedures [26]. Excessive and long questions were removed from the survey, leaving only the most valuable and unambiguous questions. A total of 1200 questionnaires were distributed and 90% of the returned questionnaires were fully completed (1080); this number of filled surveys was subjected to statistical calculations (Supplementary Materials Figure S1).

The questionnaire consisted of 27 item questions. The present paper only used questions about the vaccine against COVID-19 and the coverage rate of vaccination against COVID-19, side effects after getting the shot, type of COVID-19 vaccines, pre-vaccination stress, and attitudes towards vaccination against COVID-19 after vaccination (Supplementary Materials Table S1).

The survey covered the following questions: ‘did you take the COVID-19 vaccine?’ (yes; no, but I intend to take it in the near future; no, I do not want it) and ‘did you feel any fear or had any objections prior to the vaccination?’ (no, not at all; no, but I was a little bit unsure; I was neutral; yes, I felt a little nervous; yes, I was very nervous and anxious). The next two questions concerned the reasons for vaccinating or not vaccinating against COVID-19 of the participants: ‘what made you decide to take the COVID-19 vaccine?’ (my own health protection; my family’s health protection; it is the quickest way to going back to normal activities, i.e., being in contact with family and friends; my job and the safety of my patients; other reasons) and ‘what made you decide not to take the COVID-19 vaccine?’ (I do not believe in COVID-19 and the pandemic; I do not believe the vaccine is effective; I do not have time; I am afraid of the side effects; I do not want to take the vaccine, because I believe the vaccine was not tested long enough—there was not enough time to check and prove its effectiveness).

The survey also contained another two questions about the types of COVID-19 vaccine: ‘which vaccine were you given?’ (AstraZeneca; BioNTech/Pfizer; Moderna; Johnson & Johnson; other) and ‘did the type of vaccine influence your decision to take the vaccination?’ (no, it did not matter to me what kind of vaccine I was going to be given; yes, I delayed the decision to take the vaccination, waiting for the right vaccine; I did not have any choice and was vaccinated with a vaccine I would not personally choose). The questionnaire also includes four questions about the side effects following vaccination: ‘were there any side effects after the vaccination?’ (yes, after the first dose; yes, after the second dose; yes, after both doses; no, there were no side effects), ‘what side effects did you have after whichever dose of vaccine?’ (the symptoms were minimal, of no importance; higher temperature/fever; pain in the muscles, bones, general poor feeling; pain in the place of vaccination; headache; allergy; other symptoms); ‘how serious were the side effects after the vaccination?’ (they were minimal, of no importance; they were bad enough that I had to stay at home; they were so bad, that not only did I stay at home, but I also had to contact my GP; the side effects were severe that I had to be taken to hospital); ‘how long did you suffer the side effects after the vaccination?’ (1 day; 2–3 days; 4–7 days; longer than one week).

The participants were also asked about their attitude towards vaccines against COVID-19 after vaccination: ‘if the COVID-19 vaccination is recommended to be taken every year, should it be obligatory for the health professionals in your opinion?’ (yes, it should be obligatory; yes, it should be obligatory, if it is free of charge; no, it should be voluntary).

The remaining questions included participants’ demographic parameters (i.e., sex; age; chronic disease; occupation; major of study and year of study (among students), seniority, place of work, and place of residence (Supplementary Materials Table S1).

### 2.4. Statistical Analysis

The following statistical methods were used in the analysis: (1) t-test for mean value, (2) z test for proportions—to compare the percentage of a given category in the sub-samples (the assumptions for the z-test were examined in every case—if some of them were not met, the result is marked with an asterisk); (3) chi-squared test—to check independence between the responses of the surveyed groups (Supplementary Materials Table S2); (4) simple and multiple logistic regression to estimate the odds ratio (OR). Predictor reduction in the logistic regression was carried out using a backward elimination model selection procedure, which started with the most general model and eliminated one variable at a time until the best model was reached (i.e., when all right-side variables were statistically significant for *p* < 0.1).

Statistical calculations were carried out in (1) a Microsoft Excel 365 spreadsheet (Microsoft, Redmond, WA, USA; frequency distribution of the variables, contingency tables, z-test), (2) Gretl ver. 2020 (open-source statistical package; logit model and backward elimination procedure), and (3) STATISTICA 13.3 (StatSoft Inc., Tulsa, OK, USA; logistic regression). To check the final results, we compared the results from the logit model in GRETL with the logistic regression in STATISTICA—exponentiating the estimators from the GRETL logit model gave us the odds as well as direct estimators from the logistic regression in STATISTICA.

### 2.5. Ethical Concerns

Before collecting the survey, the respondents were informed that their responses would be confidential, that the obtained data would be used only for writing medical articles, and that taking part in this study was voluntary and anonymous. The local Bioethics Committee confirmed that, according to Polish law and Good Clinical Practice regulations, this research does not require the approval of a Bioethics Committee [27].

## 3. Results

### 3.1. Characteristic of the Study Group

The total number of respondents who took part in the study was 1,080. Participants were divided into four groups: doctors (135, various specialties), nurses and midwives (128 various specialties), and students of two majors at the medical University: 423 medical students, and 394 students of nursing and midwifery. Table 1 summarizes the demographic characteristics of the subjects: sex, age, medications for chronic diseases, place of work and institution type, and work or study experience.

All four groups were female dominated; 76.9% (830) were women and 23.1% (250) were men. The mean age was 26.8 ± 9.7 (mean age for women was 26.5 ± 10.0, for men was 27.7± 8.8; Table 1). The average age of nurses was higher than that of doctors (41.4 ± 13.1; 38.3 ± 10.6, respectively), with the opposite being the case for the students at the Medicine Faculty and in nursing and midwifery (23.6 ± 2.2 and 21.5 ± 2.1, respectively).

Of junior students, (without clinical practice), 15.6% were in the medical major (vs. 84.4% of senior students) and 52.5% were in nursing and midwifery (vs. 47.5% of senior students) (Table 1). Junior students consisted of medical students who were in years 1–3 of 6-years of study, and nursing and midwifery students who were in year 1 of 3-years of study. Among healthcare workers who had 10 years or less professional experience, 51.1% were junior doctors and 38.3% were junior nurses and midwives. A total of 48.9% of the senior group (with specialization or/and more than 10 years professional experience) were doctors, and 61.7 % were nurses and midwives. The number of senior nurses that participated in the study was approximately twice the number of junior ones.

The dominant workplace among the working respondents was the hospital (56.5%—doctors, 63.0%—nurses, 5.3%—medical students, and 14.1% nursing students), followed by specialist clinics and primary healthcare clinics (Table 1). Most of the doctors (87.4%) indicated the hospital as their most frequent workplace, but 54.8% of them reported that they work in more than one workplace. Similarly, among nurses and midwives, the hospital was the most frequently chosen answer (79.7%), but 26.6% worked in more than one workplace. Among young healthcare workers, only 5.4% of medical students and 15% of nursing and midwifery students indicated medical institutions as their workplace (mostly a hospital), and 3.3% of medical students and 6% of nursing students worked in more than one workplace (including work outside the health service in 7.8% of medical students and 12.9% of nursing and midwifery students). The predominant workplace among HCWs were institutions that were temporarily turned into COVID-19 institutions (Table 1). Such institutions employed 39.3% doctors, 37.5% nurses and midwives (37.5%), 9.9% medical major students, and 11.4% and nursing and midwifery major students. Only one third of participating healthcare workers (35.6% of doctors, 39.8% of nurses and midwives) and less than twenty percent of students at the Medical University (medical students—18.7% and 14.7% of nursing/midwifery students) reported suffering from any chronic diseases and taking medication.

### 3.2. Factors That Made the Respondents Decide to Take the COVID-19 Vaccine or Not

In the study population, 91.2% of participants were fully vaccinated against COVID-19. The most vaccinated group in this study were medical students (MS; 98.3%), followed by doctors (D; 94.8%), nursing and midwifery students (NS; 86.3%), and nurses (N; 78.9%; Table 2). Statistically significant differences were noted between D vs. N and D vs. NS (*p* < 0.001 and *p* < 0.009, respectively), and between MS vs. N and MS vs. NS (*p* < 0.001 and *p* < 0.001, respectively). There was no significant difference between NS vs. N and D vs. MS (*p* < 0.061 and *p* < 0.06, respectively; Supplementary Materials Table S2). Fifty-seven of the respondents (5.3%) did not want to be vaccinated against COVID-19. This group consisted of 9.4% NS, 11.7% N, 1.5% D, and 0.7% MS (Table 2).

In the whole study population, older healthcare workers (93.5%) were vaccinated significantly more often than younger ones (87.2%; *p* < 0.001; Supplementary Materials Table S3). However, this difference between juniors and seniors within individual study groups (except medical students) was not statistically significant.

The *p*-value for the chi-square test of independence between the groups of healthcare workers and the decision to vaccinate showed that the reasons why the respondents were vaccinated against COVID-19 differed statistically between D vs. MS (*p* = 0.001), D vs. NS (*p* = 0.004), N vs. MS (*p* = 0.054), and NS vs. MS (*p* = 0.001; Supplementary Materials Table S2). More details for each of the reasons for vaccinating against COVID-19 and refusal are presented in Table 2 and Supplementary Materials Table S3.

Protecting one’s own health was the most common reason for both senior doctors and nurses to be vaccinated against COVID-19 (41.8% vs. 36.4%, respectively). In contrast, medical students (both junior and senior) most often reported that protecting the health of their relatives was the most important factor prompting vaccination against COVID-19 (37.3% and 38.2%, respectively; Supplementary Materials Table S3). For junior doctors and nurses and both groups of nursing students, the above two reasons for vaccination were of comparable importance.

Another important reason for the decision to vaccinate among doctors, especially from the senior group, and senior nurses, was the safety of their patients (Table 2 and Supplementary Materials Table S3). On the other hand, this reason was less important than the possibility of returning back to life before the pandemic for the remaining groups of respondents (Table 2)—as they perceived vaccination as the quickest way to go back to normal activities, e.g., meeting with their family and friends. Among the students who selected ‘others’, the most common explanation was the fear of not being able to participate in clinical classes or an internship due to their non-vaccination status.

In total, the largest group of unvaccinated people were nurses (by summing up respondents delaying vaccination and refusing to vaccinate at all; 21.1%), followed by NS (13.7%; Table 2). In contrast, there were only isolated cases of unvaccinated doctors and medical students, including only two doctors (1.5%) and three medical students (0.7%) who definitely did not want to be vaccinated (Table 2), and five doctors (3.7%) and four medical students (0.9%) delaying vaccination.

The most common reasons that were given by nurses and medical students against COVID-19 vaccination were: “the vaccine wasn’t tested long enough” followed by fear of post-vaccination complications and lack of faith in vaccine efficacy (Table 2). The same reasons were mentioned by NS, although in a different order. Lack of time also played an important role in doctors not receiving vaccinations. Some stated other reasons, listing previous COVID-19 disease as their reason for not vaccinating.

### 3.3. Pre-Vaccination Stress or Any Other Objections Prior to the Vaccination against the COVID-19 in Vaccinated Healthcare Professionals

Pre-vaccination stress varied significantly between occupational groups (*p* < 0.001)—except for nurses and nursing students (*p* = 0.513; Supplementary Materials Table S2). Detailed statistical data on this issue for individual groups of respondents are presented in Table 3 and Supplementary Materials Table S4.

More than two-thirds of the surveyed doctors and medical students declared that they had no fear before vaccination against COVID-19 (Table 3). The group of senior healthcare workers/students reported feeling no fear before getting the vaccine against COVID-19 more often than the junior ones, although statistically significant differences were noted only for medical students (Supplementary Materials Table S4). On the other hand, a complete lack of fear of vaccination was declared by only about 40% of nurses and midwives, as well as of nursing and midwifery students. Meanwhile, while in total only about 10% of physicians and medical students expressed slight or serious concerns about vaccination, this opinion concerned three times more N and NS (Table 3). Additionally, high stress before the COVID-19 shot was noted the most commonly among junior NS—11.5% and senior nurses −9.5% (Supplementary Materials Table S4).

### 3.4. The Kinds of Vaccine against COVID-19 and Healthcare Professionals

Due to the availability of vaccines at the time, most healthcare workers were vaccinated with either the AstraZeneca or BioNTech/Pfizer vaccine, with the latter predominating (Table 4). A total of 84.1% of subjects were vaccinated with BioNTech/Pfizer’s vaccine, including doctors (96.1%) and nurses and midwives (91.1%). The AstraZeneca vaccine was used for the vaccination of 28.8% NS and 7% MS. Only a small group of participants were vaccinated with the COVID-19 vaccines of other pharmaceutical companies such as Moderna or Johnson & Johnson; these cases consisted of healthcare workers who decided to vaccinate relatively late, or who were waiting until these particular vaccines were available in Poland.

For almost 90% of doctors, it did not matter which COVID-19 vaccine they received, and waiting for the “right” vaccine was observed only in isolated cases (Table 4). Additionally, the majority of medical students did not have a preference for the type of vaccine (76.5%), and even if so, they agreed to be vaccinated against their personal preference (14%). Less than 10% of medical students withheld vaccination due to waiting for a specific type of vaccine. While in the case of N and NS, over three times more respondents delayed the decision to take the vaccination while waiting for another kind of vaccine (Table 4). About half of the N and NS respondents were indifferent to which vaccine they received.

### 3.5. Any Side Effects after Taking the COVID-19 Vaccine

The occurrence, type, and severity of adverse post-vaccination reactions (APVRs) were assessed only for the BioNTech/Pfizer and the AstraZeneca vaccines, which were applied to a sufficient number of respondents in the study group in order to perform statistical analyses and draw reliable conclusions. The remaining Johnson & Johnson and Moderna vaccines were excluded from the statistical comparison due to the very low vaccine coverage ratio.

One-fourth of the study’s population (28%) that were vaccinated with the BioNTech/Pfizer vaccine and 11% with AstraZeneca did not have any side effects after being vaccinated against COVID-19 (Table 5). In one-third of the vaccinated HCWs, side effects occurred after both doses of the vaccine, regardless of its type. However, for the AstraZeneca vaccine, one in two subjects reported APVRs only for the first dose. In contrast, for the BioNTech/Pfizer vaccine, the second dose was more likely to produce side effects than the first. In the study population, the most commonly reported adverse events after vaccination were in the following order: pain at the place of vaccination (on the forearm), pain in the muscles, bones, general poor feeling, and fever. Regardless of the vaccine, these side effects affected about 1/4 of the population (Table 5). Fourteen to nineteen percent of those vaccinated also reported a headache. 

More than 50% of the people vaccinated with BioNTech/Pfizer vaccine considered their symptoms mild, compared to 13% of the AstraZeneca recipients (Table 5). On the other hand, 73% who were vaccinated with the AstraZeneca vaccine, and 43% with BioNTech/Pfizer had to stay at home the day after receiving the dose due. Only a few respondents had such serious side effects that they not only decided to stay at home, but also contacted their GP. This was more common for those vaccinated with the AstraZeneca vaccine. None of the vaccinated participants in the above study were hospitalized, and none required medical intervention for APVR after COVID-19 vaccination. Regardless of the vaccine used, a comparable number of respondents indicated that their APVR lasted 1 day (43–49%) or 2–3 days (44%; Table 5). Only 1% of those vaccinated with the AstraZeneca vaccine and 2% of those vaccinated with the BioNTech/Pfizer vaccine suffered from APVRs for more than a week.

Table 6 compares the incidence of APVR with different demographic factors, fear of vaccination, and the type of vaccine used (AstraZeneca and BioNTech/Pfizer vaccine). Apart from the type of vaccine used, which had the greatest impact on the incidence of side effects, two factors increased the risk of developing APVR: a history of chronic disease, and a significant fear of vaccination. None of the demographic factors influenced the severity of APVRs. The only factor that played a statistically significant role (in the multivariate version of the logistic regression) was the type of vaccine (Table 7). In contrast, in addition to the type of vaccine, factors such as the age of the individual and their fear of vaccination had statistical significance on the duration of the adverse effects (Table 8). For participants under 27 years of age, they were less likely to have a reaction lasting longer than 4 days, compared to the elderly, who were twice as likely to have an extended adverse reaction. Longer APVR times were also found more often for people who were afraid of vaccination or who had only slight doubts about vaccination compared to the group that did not fear vaccination against COVID-19 at all (Table 8).

By assessing the differences between study participants in terms of their seniority, seniors were statistically more likely to support mandatory vaccination than juniors (46.4 vs. 35.9%), whilst a significantly lower percentage of seniors favored voluntary vaccination compared to juniors (24.4 vs. 33.5%; Supplementary Materials Table S5). On the other hand, in relation to nurses and midwives, as many as 49.4% of seniors in these professions were in favor of voluntary vaccination, compared to 42.6% of juniors in favor of this solution.

Significantly, participants who were vaccinated with the BioNTech/Pfizer vaccine more often recommended the introduction of obligatory vaccinations against COVID-19 for health professionals than those vaccinated with the AstraZeneca vaccine (48% and 30%, respectively, *p* < 0.001; Table 5). On the other hand, individuals vaccinated with AstraZeneca most often advocated for voluntary COVID-19 vaccinations for health professionals, regardless of payment (39%).

### 3.6. Attitude of Participants towards Vaccination against COVID-19

In our study, approximately 50% of doctors and medical students, but only approximately 33% of nurses and midwives and nursing and midwifery students responded that they supported the decision for mandatory vaccination against COVID-19 among HCWs (Table 9). Significant differences were also noted between D vs. N and D vs. NS (*p* < 0.005; *p* < 0.001, respectively) and between MS vs. N and NS vs. NS (*p* < 0.001 and *p* < 0.001, respectively). In addition, nearly half of N and one-third of NS were in favor of voluntary vaccination. On the other hand, for approximately 30% of respondents, the deciding factor on which they made their recommendation was the payment for this vaccination. To these respondents, this was the necessary condition for making COVID-19 vaccination mandatory.

## 4. Discussion

In the present study, many of the important issues around vaccination against COVID-19 were investigated. Based on the available publications and the best knowledge of the authors, this is the first study that presents the vaccination coverage level against COVID-19 among different groups of healthcare workers and two majors of medical students six months after the introduction of vaccinations in Poland. The immunization coverage was 94.8% among doctors, 78.9% in nurses and midwives, 98.3% in medical students, and 86.3% in nursing and midwifery students.

Our study confirms the findings of a pre-vaccination survey carried out in Slovenia, which showed that a higher percentage of physicians and medical students intended to get vaccinated when compared to nurses [28]. Additionally, in Turkey, the risk of not getting vaccinated/late vaccination was significantly higher in nurses/midwives than in doctors [29], while among French HCWs, the highest percentages of those wanting to become vaccinated were physiotherapists (95.8%) and doctors (92.1%), followed by pharmacists (88.8%), and the lowest were nurses (64.7%) [21]. Moreover, a survey conducted by the American Nurses Foundation published in October 2020 suggested that only 34% of nurses were willing to be vaccinated with the COVID-19 vaccine, while 36% refused and 31% were unsure [30].

On the other hand, the level of immunization among nurses in Poland, although slightly lower than that obtained in doctors and medical students, was still very high. This figure was higher than the threshold for a population to gain herd immunity. This is a significantly better result compared to the annual vaccination coverage of influenza among HCWs in Poland (5–10% for nurses and about 20% for doctors) [31]. Moreover, the vaccination rate in our study population was much higher than the COVID-19 vaccine acceptance rate in the Polish general public among adults (56.9%) [32]. Additionally, more surveyed medical students in Poland (92%) than non-medical students (59.4%) indicated a desire to get vaccinated against SARS-CoV-2 [33]. Similar to our study, HCWs in Qatar showed a higher rate of vaccine acceptance when compared to other non-health-related disciplines [22].

Moreover, the vaccination rate of HCWs in Poland was found to be significantly higher than that predicted before the vaccine became available. For example, the declaration rate ranged from 27.7% for HCWs in The Democratic Republic of the Congo (March and April 2020) [34]; 44.2% for health science and medicine students [35], 52.0% for HCWs [23], and 61.8% for GPs in Malta (September 2020) [36]; 61,1% for nurses and 78.1% for doctors in Israel (March and April 2000) [24]; 63% for nurses in Hong Kong (March to April 2020) [37]; 76.9% for HCWs in France (March to July 2020) [21]; to 86.1% for university students in Italy (June 2020) [38]. A study of the intentions of Polish HCWs to be vaccinated was completed in autumn of 2020 and showed that less than 70% of respondents would like to be vaccinated against COVID-19 [39]. Likewise, higher actual vaccination coverage among health professionals could therefore be expected in other countries, due to the higher immunization coverage in the general population than the earlier forecasts for HCWs [21,23,36,37,38,40]. On the other hand, only a slight increase in the willingness to get vaccinated against COVID-19 was reported in the general population in Poland 2 months after the start of the vaccination program compared to the period before the commencement of the vaccination program; in both cases about 50% [41]. This level of vaccination coverage in Poland was achieved in August 2021, 4 months from the time when vaccination became available to all citizens [42]. Since then, the increase in vaccination coverage has been slow, and vaccinations in the last 3 months have mostly been taken by those who decided to take the 3rd dose, not those who had not been vaccinated so far (except for the age group 5–11, which was only approved for vaccination at the end of December 2021) [40,42].

Several factors may have an influence on HCW attitudes toward COVID-19 vaccination in Poland. The most important being easy access to vaccines and no payment for them. The vaccination procedure for HCWs and medical students in Poland was organized by hospitals, clinics, and universities. It was sufficient to express a willingness to be vaccinated by e-mail or by phone, and you would be informed about the time and place of vaccination afterwards. Additionally, although vaccination against COVID-19 is voluntary for the time being, university authorities and directors of health centers have introduced educational programs, invoked responsibility for patients, and applied some pressure to achieve the highest possible level of vaccination among HCWs. Additionally, a group of students in our study mentioned that one of the reasons for undergoing vaccination was due to concerns about the possibility of participating in clinical classes, internships, or attempts to graduate from medical studies in the event of not being vaccinated. In addition, around 30% of primary healthcare workers in the study postulated that vaccination against COVID-19 should be mandatory among healthcare professionals, but only if vaccination is free. This is particularly important in the context of the announcement of obligatory vaccination against COVID-19 of HCWs in Poland in the near future (March 2022). Some European countries such as France, Greece, Italy, and Hungary already made COVID vaccination mandatory for healthcare workers last year [43,44]. Implementing this policy is a response to a social need for the protection of public health, and above all as a defense for patients, for whom HCWs have a specific position of guaranteed protection and trust. Simultaneously, to obtain a sufficient level of immunization in the general population, some countries have chosen to adopt a control measure called "vaccine passports" [45]. The pass is awarded after a full vaccination cycle or recent recovery from COVID-19, and it allows access to different public places such as cinemas or restaurants and to travelling. In Poland, since July 1, digital international COVID-19 immunization certificates have been adopted, but they are only used during trips abroad, because no regulations were introduced that would allow them to be practically enforced inside the country. On the other hand, some reported findings suggest that vaccine passports resulted in autonomy frustration and might have detrimental effects on people’s motivation to get vaccinated, making them less likely to sign-up for a "booster" dose of the vaccine [45,46].

The two most important reasons for vaccination found among HCWs in our study were personal and familial protection. The same reasons were also key to the decision to vaccinate against COVID-19 for HCWs in other studies [47,48]. Moreover, an important motivation for the large group of respondents to our study was a general risk reduction and the prospective possibility of lifting personal restrictions. This factor was also very significant among those vaccinated in the 30–55 age group in Switzerland [49].

On the other hand, there was a very low percentage of unvaccinated participants in our study. These individuals can be divided into two groups according to the definitions of SAGE experts (the Strategic Advisory Group of Experts on immunization): persons who delay taking the vaccine (3.5%) and those who refuse vaccination despite the availability of vaccines (5.3%) [50,51]. However, this definition does not take into account the concept of stress or fear of vaccination, although it indirectly concerns the trust in vaccination safety, which is an important element preventing willingness to accept the vaccine. In this study, every fourth N and NS experienced fear or objections before their vaccination. In the present study, most often both unvaccinated or still-hesitating subjects reported that they were afraid of the side effects and that the vaccine was not tested long enough. Those reasons cannot overwhelm the global efforts to control the COVID-19 pandemic by preventing the achievement of a high level of vaccination (at least 70% of immune individuals to obtain herd immunity and stop the spread of the virus in the population, and to protect people who cannot be vaccinated for medical reasons) [52,53]. Before each vaccination, the doctor collects an interview from the patient regarding medical contraindications for vaccination, but no one asks about anxiety before vaccination. This feeling of uncertainty about how one will feel after vaccination causes a build-up of stress and a reluctance to continue vaccination in the future.

The three most common reasons for not getting vaccinated in our study were the rapid completion of clinical trials, doubt concerning the efficacy of the vaccine, and fear of side effects. Our findings support prior studies that also are also concerned with healthcare professionals [29,54]. Skeptical attitudes towards new vaccines are mainly due to a perceived lack of testing for vaccine safety and efficacy [28,55]. The accelerated release of vaccines under emergency use authorization (EUA) rather than full FDA approval could be important causes for skepticism [56]. Meanwhile, the occurrence of pre-vaccination stress among participants was one of the factors that significantly influenced the occurrence of APVRs and their duration (Table 6 and Table 7). Accurate information about possible side effects and how to deal with them can reduce the feeling of fear of vaccination, and here is the main role of HCWs—to know about vaccination and inform patients when they qualify for vaccination. The attitudes of HCWs related to COVID-19 vaccination are particularly important for enhancing vaccination uptake in general populations [57].

More than two-thirds of vaccinated participants in the study reported side effects after receiving at least one dose of vaccine against COVID-19. An adverse event after the first dose of AstraZeneca was significantly more frequent than after the BioNTech/Pfizer vaccine, and inversely after receiving the second dose of these vaccines (*p* < 0.001). The respondents more often rated the side effects after vaccination with the BioNTech/Pfizer vaccine (52%) as minimal vs. vaccination with the AstraZeneca vaccine (13%; *p* < 0.05). Severe post-vaccination adverse events that forced individuals to stay home occurred more frequently after the AstraZeneca vaccine, and side effects were severe enough to force them to contact a GP three times more often than after vaccination with the BioNTech/Pfizer vaccine (*p* < 0.05). These data translate into observations made in the general population. According to the GIS (Chief Sanitary Inspectorate) in Poland, by 15 April 2021 adverse post-vaccination reactions following the AstraZeneca vaccine were reported, of which 25 were very severe, 324 serious, and 2708 mild—while there was a total of 2576 adverse vaccine reactions following the BioNTech/Pfizer vaccine, including 101 severe, 397 serious, and 2078 mild [11]. Side effects associated with COVID-19 vaccines by AstraZeneca and BioNTech/Pfizer have been also reported by 60% of those vaccinated in Saudi Arabia [58]. It has been reported that the side effects associated with the COVID-19 vaccine are mild to moderate. Similar to our results, where less than 6% HCWs required consultation with a doctor, only 3% of the participants needed to see a doctor due to the side effects of the vaccines in the study of Alhazmi et al. [58].

It can therefore be assumed that encouragement and support from employers in the form of a day or two off in case of feeling unwell after vaccination could prove to be an additional factor in convincing individuals to vaccinate. The benefits of vaccinating HCWs are much greater, even if they are given 1–2 days off after vaccination, than of unvaccinated HCWs still working with patients—especially because a significant number of people participating in the presented study declare several workplaces and, if infected, they pose an even greater threat for their patients.

It should also be considered that most APVRs were mild and transient, and vaccination, like any medical procedure, carries some risks—and in the case of mass vaccinations among populations of all ages, with various medical conditions, it is not surprising that there have also been noted very serious complications, such as thrombotic complications, post-vaccination heart failure, or anaphylactic shock. However, these cases are very rare. On the one hand, adverse events including anaphylaxis cases occurred in individuals with a documented history of hypersensitivity after COVID-19 vaccination [59], but on the other hand, according to Nittner-Marszalska et al., 2021 [60], vaccination with the BioNTech/Pfizer anti-COVID-19 vaccine was well tolerated by both persons with and without a history of allergy/anaphylaxis. The vast majority of the reported symptoms of hypersensitivity were localized. Furthermore, these cases, although incidental, should not be concealed, because concealing any information may only lead to the building of conspiracy theories, which will cause all the more fear and destroy confidence in vaccinations [61]. This is all the more important as the current epidemiological situation may suggest that further doses of the vaccine will be required before the global COVID-19 pandemic is brought under control.

Additionally, some reports have revealed changes over time relative to vaccination against SARS-CoV-2 in society, which could be positively related to the number of individuals who have been already vaccinated. As the number of people vaccinated increases and the number of reports about the efficacy and safety of the COVID-19 vaccine increases, the acceptance rate for vaccines also increases [62].

### Limitations of This Study

The study has several limitations. A major limitation of our study is the time of survey release; our detailed analysis of post-vaccination effects could only concern two of the four vaccination type preparations available on the market, because, at the time of collecting the questionnaires, the remaining vaccines were not common and too low a percentage of HCWs were vaccinated with them for the results to be analyzed in detail. Most of the respondents who participated in the study got vaccinated and support vaccination. However, it is likely that unvaccinated staff may have been more reluctant to participate in the study and did not complete the questionnaires (out of 1200 questionnaires that were distributed, 1080 were completed).

## 5. Conclusions

Our findings demonstrate a very high rate of vaccination against COVID-19 among HCWs in Poland, and a high understanding of the desirability and necessity of universal vaccinations. However, taking into account that vaccines and the possible APVRs after them arouse a certain fear even among HCWs educated in this regard, it is worth taking measures that would eliminate pre-vaccination stress in order to obtain a high vaccination rate in the entire population—repeatedly if vaccination against COVID-19 would have to be a permanent feature of our annual vaccination schedule. Vaccine hesitancy continues to be a significant concern during the COVID-19 pandemic and needs to be addressed through multiple avenues based on scientific evidence, because even medical personnel can be susceptible to misinformation. Reducing distrust regarding COVID-19 vaccination and increasing the willingness to take the vaccine among HCWs may result in an increase in vaccination acceptance among the general population, which closely monitors how HCWs behave in this matter. The attitudes of HCWs are particularly important for the acceptance of vaccination in general populations, as people are more likely to get vaccinated if recommended to do so by their HCW. The approach to vaccination of nurses and nursing students is particularly important because these HCWs spend most of their time in direct contact with patients and play a crucial role in the process of vaccination. Moreover, the benefit of optimizing a vaccination program is the promotion of vaccinations at universities and hospitals, and therefore, medical authorities should focus on giving further reliable knowledge regarding the COVID-19 pandemic, and persuade or even require HCWs to take the COVID-19 vaccine.

## Figures and Tables

**Table 1:** Socio-demographics characteristics of respondents in four healthcare worker groups.

	Doctors (D) n = 135 (12.5%)	Nurses and Midwives (N) n = 128 (11.8%)	Medical Students (MS) n = 423 (39.2%)	Nursing and Midwifery Students (NS) n = 394 (36.5%)	Total n = 1080 (100%)
Sex					
Female	71 (52.6%)	121 (94.5%)	264 (62.4%)	374 (94.9%)	830 (76.9%)
Male	64 (47.4%)	7 (5.5%)	159 (37.6%)	20 (5.1%)	250 (23.1%)
Total	135 (100.0%)	128 (100.0%)	423 (100.0%)	394 (100.0%)	1080 (100.0%)
Age					
Age, mean (SD)	38.3 (10.6)	41.4 (13.1)	23.6 (2.2)	21.5 (2.1)	26.8 (9.7)
19–26 y	5 (3.7%)	28 (21.9%)	402 (95.1%)	380 (96.5%)	815 (75.5%)
≥27 y	130 (96.3%)	100 (78.1%)	20 (4.7%)	10 (2.5%)	260 (24.1%)
No data	0 (0.0%)	0 (0.0%)	1 (0.2%)	4 (1.0%)	5 (0.5%)
Total	135 (100.0%)	128 (100.0%)	423 (100.0%)	394 (100.0%)	1080 (100.0%)
Year of undergraduate study/Year of work					
Junior	69 (51.1%)	49 (38.3%)	66 (15.6%)	207 (52.5%)	391 (36.2%)
Senior	66 (48.9%)	79 (61.7%)	357 (84.4%)	187 (47.5%)	689 (63.8%)
Total	135 (100.0%)	128 (100.0%)	423 (100.0%)	394 (100.0%)	1080 (100.0%)
Chronic disease					
Yes	48 (35.6%)	51 (39.8%)	79 (18.7%)	58 (14.7%)	236 (21.9%)
No	87 (64.4%)	74 (57.8%)	344 (81.3%)	335 (85.0%)	840 (77.8%)
No data	0 (0.0%)	3 (2.3%)	0 (0.0%)	1 (0.3%)	4 (0.4%)
Total	135 (100.0%)	128 (100.0%)	423 (100.0%)	394 (100.0%)	1080 (100.0%)
Place working (multiple choice)					
Hospital	118 (56.5%)	102 (63.0%)	23 (5.3%)	59 (14.1%)	302 (24.6%)
Outpatient clinic	30 (14.4%)	19 (11.7%)	10 (2.3%)	13 (3.1%)	72 (5.9%)
Specialist Clinic	46 (22.0%)	24 (14.8%)	6 (1.4%)	7 (1.7%)	83 (6.8%)
Hospital Emergency Ward	11 (5.3%)	6 (3.7%)	1 (0.2%)	8 (1.9%)	26 (2.1%)
Emergency medical Services	3 (1.4%)	4 (2.5%)	1 (0.2%)	3 (0.7%)	11 (0.9%)
I work outside the health service	1 (0.5%)	4 (2.5%)	33 (7.6%)	51 (12.2%)	89 (7.3%)
I do not work yet, I am still a student	0 (0.0%)	3 (1.9%)	363 (83.1%)	277 (66.3%)	643 (52.4%)
Total	209 (100.0%)	162 (100.0%)	437 (100.0%)	418 (100.0%)	1226 (100.0%)
Daily contact with COVID-19 patients during the pandemic					
Yes, I worked in a COVID-19 institution	53 (39.3%)	48 (37.5%)	42 (9.9%)	45 (11.4%)	188 (17.4%)
No, but many COVID-19 patients were treated in my place of work	50 (37.0%)	40 (31.3%)	32 (7.6%)	57 (14.5%)	179 (16.6%)
No, but sometimes patients turned out to be infected with COVID-19	28 (20.7%)	36 (28.1%)	199 (47.0%)	133 (33.8%)	396 (36.7%)
No contact with COVID-19 patients	4 (3.0%)	3 (2.3%)	150 (35.5%)	158 (40.1%)	315 (29.2%)
No data	0 (0.0%)	1 (0.8%)	0 (0.0%)	1 (0.3%)	2 (0.2%)
Total	135 (100.0%)	128 (100.0%)	423 (100.0%)	394 (100.0%)	1080 (100.0%)

Junior—first years of study (1–3 for MS, 1 for NS)/up to 10 years, inclusive, of work in the profession; Senior—last years of study (4–6 years for MS, 2–3 years for NS)/more than 10 years of work in the profession; SD-standard deviation.

**Table 2:** Differences (%) between four groups of healthcare workers (doctors, nurses and midwives, medical students, and nursing students) and reasons for vaccination or non-vaccination against COVID-19.

	D (n = 135)	N (n= 128)	MS (n = 423)	NS (n = 394)	Total (n = 1080)	D vs. N	D vs. MS	D vs. NS	N vs. MS	N vs. NS	NS vs. MS
Vaccinated	128 (94.8%)	101 (78.9%)	416 (98.3%)	340 (86.3%)	985 (91.2%)						
Reasons for vaccination (multiple choice)											
My own protection	94 (39.2%)	57 (32.8%)	233 (30.5%)	172 (31.2%)	556 (32.1%)	0.182	0.012	0.029	0.553	0.692	0.785
My family’s protection	71 (29.6%)	51 (29.3%)	291 (38.0%)	169 (30.6%)	582 (33.6%)	0.952	0.017	0.771	0.031	0.744	0.005
Going back to normality	31 (12.9%)	31 (17.8%)	143 (18.7%)	109 (19.7%)	314 (18.1%)	0.169	0.039	0.021	0.788	0.574	0.632
The safety of my patients	44 (18.3%)	32 (18.4%)	93 (12.2%)	86 (15.6%)	255 (14.7%)	0.988	0.015	0.337	0.029	0.381	0.074
Other	0 (0.0%)	3 (1.7%)	5 (0.7%)	16 (2.9%)	24 (1.4%)	0.042 *	0.210 *	0.008 *	0.166 *	0.398 *	0.001 *
Total	240 (100%)	174 (100%)	765 (100%)	552 (100%)	1731 (100%)						
Not yet vaccinated	5 (3.7%)	12 (9.4%)	4 (0.9%)	17 (4.3%)	38 (3.5%)						
Reasons for not yet vaccinating (multiple choice)											
I do not believe in COVID-19 and the pandemic	0 (0.0%)	1 (6.7%)	0 (0.0%)	1 (3.0%)	2 (3.5%)	0.561 *	-	0.696 *	0.603 *	0.562 *	0.726 *
I do not believe the vaccine is effective	0 (0.0%)	2 (13.3%)	1 (25.0%)	10 (30.3%)	13 (22.8%)	0.401 *	0.274 *	0.160 *	0.577 *	0.215 *	0.828 *
I do not have time	2 (40.0%)	1 (6.7%)	0 (0.0%)	3 (9.1%)	6 (10.5%)	0.087 *	0.195 *	0.065 *	0.603 *	0.779 *	0.533 *
I am afraid of the side effects	0 (0.0%)	2 (13.3%)	1 (25.0%)	11 (33.3%)	14 (24.6%)	0.401 *	0.274 *	0.134 *	0.128 *	0.155 *	0.514 *
Vaccine was not tested long enough	0 (0.0%)	5 (33.3%)	2 (50.0%)	8 (24.2%)	15 (26.3%)	0.153 *	0.116 *	0.223 *	0.754 *	0.514 *	0.97 *
Other	3 (60.0%)	4 (26.7%)	0 (0.0%)	0 (0.0%)	7 (12.3%)	0.193 *	0.100 *	<0.001 *	0.261 *	0.003 *	-
Total	5 (100%)	15 (100%)	4 (100%)	33 (100%)	57 (100%)						
Do not want vaccination	2 (1.5%)	15 (11.7%)	3 (0.7%)	37 (9.4%)	57 (5.3%)						
Reasons for reluctance to vaccinate (multiple choice)											
I do not believe in COVID-19 and the pandemic	0 (0.0%)	4 (16.7%)	1 (33.3%)	2 (3.6%)	7 (8.2%)	0.536 *	0.429 *	0.787 *	0.490 *	0.045 *	0.026 *
I do not believe the vaccine is effective	0 (0.0%)	6 (25.0%)	0 (0.0%)	11 (19.6%)	17 (20.0%)	0.428 *	-	0.489 *	0.336 *	0.593 *	0.398 *
I do not have time	0 (0.0%)	0 (0.0%)	0 (0.0%)	4 (7.1%)	4 (4.7%)	-	-	0.697 *	-	0.183 *	0.633 *
I am afraid of the side effects	0 (0.0%)	6 (25.0%)	0 (0.0%)	19 (33.9%)	25 (29.4%)	0.428 *	-	0.319 *	0.336 *	0.432 *	0.226 *
Vaccine was not tested long enough	2 (100%)	8 (33.3%)	1 (33.3%)	16 (28.6%)	27 (31.8%)	0.075 *	0.233 *	0.036 *	1.000 *	0.671 *	0.860 *
Other	0 (0.0%)	0 (0.0%)	1 (33.3%)	4 (7.1%)	5 (5.9%)	-	0.429 *	0.697 *	0.008 *	0.183 *	0.118 *
Total	2 (100%)	24 (100%)	3 (100%)	56 (100%)	85 (100%)						

D—doctors, N—nurses and midwives, MS—medical Students, NS—nursing and midwifery students; *—low-credibility data due to the low size of the compared groups; dash indicates that there is no data in a given group for statistical analysis.

**Table 3:** Stress, fear, or any objections prior to vaccination against COVID-19 in four groups of vaccinated healthcare workers.

	D	N	MS	NS	Total	D vs. N	D vs. MS	D vs. NS	N vs. MS	N vs. NS	NS vs. MS
No, not at all	86 (67.2%)	42 (41.6%)	285 (68.8%)	124 (36.5%)	537 (54.6%)	<0.001	0.725	<0.001	<0.001	0.352	<0.001
No, I was a little bit unsure	17 (13.3%)	20 (19.8%)	69 (16.7%)	96 (28.2%)	202 (20.5%)	0.184	0.360	0.001	0.455	0.092	<0.001
I was neutral	11 (8.6%)	2 (2.0%)	3 (0.7%)	9 (2.6%)	25 (2.5%)	0.033 *	<0.001 *	0.005 *	0.249 *	0.706 *	0.036 *
Yes, I felt a little nervous	9 (7.0%)	29 (28.7%)	50 (12.1%)	84 (24.7%)	172 (17.5%)	<0.001 *	0.110 *	<0.001 *	<0.001	0.418	<0.001
Yes, I was very nervous and anxious	5 (3.9%)	8 (7.9%)	7 (1.7%)	27 (7.9%)	47 (4.8%)	0.194 *	0.137 *	0.124 *	0.001 *	0.995*	<0.001 *
Total	128 (100%)	101 (100%)	414 (100%)	340 (100%)	983 (100%)						

D—doctors, N—nurses and midwives, MS—medical Students, NS—nursing and midwifery students; *—low-credibility data due to the low size of the compared groups.

**Table 4:** The decision to vaccinate against COVID-19 and the type of vaccine (AstraZeneca, BioNTech/Pfizer, Moderna, Johnson & Johnson) in four groups of vaccinated healthcare workers.

	D	N	MS	NS	Total	D vs. N	D vs. MS	D vs. NS	N vs. MS	N vs. NS	NS vs. MS
Vaccination type											
AstraZeneca	3 (2.3%)	5 (5.0%)	29 (7.0%)	98 (28.8%)	135 (13.7%)	0.287 *	0.052 *	0.052 *	0.463 *	<0.001 *	0.041 *
BioNTech/Pfizer	123 (96.1%)	92 (91.1%)	378 (90.9%)	235 (69.1%)	828 (84.1%)	0.118 *	0.056 *	0.056 *	0.944 *	<0.001 *	<0.001 *
Moderna or Johnson & Johnson	2 (1.6%)	4 (4.0%)	9 (2.2%)	7 (2.1%)	22 (2.2%)	0.261 *	0.673 *	0.673 *	0.301 *	0.283 *	<0.001 *
Total	128 (100%)	101 (100%)	416 (100%)	340 (100%)	985 (100%)						
Influence of vaccine type on vaccination decision											
No. it did not matter	111 (86.7%)	45 (44.6%)	316 (76.5%)	183 (53.8%)	655 (66.7%)	<0.001	<0.001 *	<0.001	<0.001	0.102	<0.001
Yes. I waited for the “right” vaccine	8 (6.3%)	36 (35.6%)	39 (9.4%)	96 (28.2%)	179 (18.2%)	<0.001 *	<0.001 *	<0.001 *	<0.001	0.154	<0.001
I did not have any choice and was vaccinated with a vaccine I would not personally choose	9 (7.0%)	20 (19.8%)	58 (14.0%)	61 (17.9%)	148 (15.1%)	0.004 *	<0.001 *	0.003 *	0.149	0.672	0.145
Total	128 (100%)	101 (100%)	413 (100%)	340 (100%)	982 (100%)						

D—doctors, N—nurses and midwives, MS—medical Students, NS—nursing and midwifery students; *—low-credibility data due to the low size of the compared groups.

**Table 5:** Side effects after vaccination against COVID-19 among the vaccinated healthcare workers.

	Vaccine Type		
	AstraZeneca	BioNTech/Pfizer	*p*
Influence of vaccine type on vaccination decision			
No, it did not matter	47 (35%)	97 (12%)	<0.001
Yes, I waited for the “right” vaccine	67 (50%)	576 (70%)	<0.001
I did not have any choice and was vaccinated with a vaccine I would not personally choose	21 (16%)	152 (18%)	0.422
Total	135 (100%)	825 (100%)	
Side effects after the vaccination			
Yes, after the first dose	65 (48%)	121 (15%)	<0.001
Yes, after the second dose	12 (9%)	211 (25%)	<0.001
Yes, after both doses	43 (32%)	261 (32%)	0.939
No, there were no side effects	15 (11%)	235 (28%)	<0.001
Total	135 (100%)	828 (100%)	
What side effects? (multiple choice)			
The symptoms were minimal	7 (2%)	112 (8%)	<0.001
Fever	95 (26%)	299 (21%)	0.043
Pain in the muscles, bones; general poor feeling	95 (26%)	354 (25%)	0.650
Pain in the place of vaccination	85 (24%)	411 (29%)	0.042
Headache	68 (19%)	200 (14%)	0.031
Allergy	0 (0%)	10 (1%)	0.109
Other	9 (3%)	34 (2%)	0.929
Total	359 (100%)	1420 (100%)	
Intensification of side effects			
Of no importance	16 (13%)	310 (52%)	<0.001
Bad enough that I had to stay at home	88 (73%)	256 (43%)	<0.001
I stayed at home and I also had to contact my GP	16 (13%)	25 (4%)	<0.001
Total	120 (100%)	591 (100%)	
How long did you suffer the side effects?			
1 day	52 (43%)	291 (49%)	0.252
2–3 days	53 (44%)	262 (44%)	0.998
4–7 days	14 (12%)	28 (5%)	0.003
Longer than one week	1 (1%)	12(2%)	0.374
Total	120 (100%)	593 (100%)	
Should the vaccine be obligatory for the health professionals			
Obligatory	40 (30%)	392 (48%)	<0.001
Obligatory, if it is free of charge	41 (31%)	251 (31%)	0.995
Voluntary	52 (39%)	172 (21%)	<0.001
Total	133	815	

**Table 6:** Associations between sex, age, chronic disease, seniority, fear of vaccination, COVID infection, the type of vaccine, and the occurrence of adverse reactions after vaccination.

	With APVR	Without APVR	Univariate Logistic Regression		Multivariate Logistic Regression	
	N (%)	N (%)	OR 95%CI	*p*	OR 95%CI	*p*
Total	727 (73.8%)	258 (26.2%)				
Sex						
Male	164 (69.2%)	73 (30.8%)	Ref			
Female	563 (75.3%)	185 (24.7%)	1.35 (0.98–1.87)	0.064		
Age						
≥27 y	161 (70.6%)	67 (29.4%)	Ref.			
19–26 y	566 (74.8%)	191 (25.2%)	1.23 (0.89–1.71)	0.212		
Chronic disease						
No	557 (72.3%)	213 (27.7%)	Ref.			
Yes	166 (78.7%)	45 (21.3%)	1.40 (0.97–2.02)	0.070	1.46 (1.01–2.11)	0.047
Seniority						
Junior	246 (72.1%)	95 (27.9%)	Ref			
Senior	962 (74.7%)	326 (25.3%)	1.230.891.71	0.211		
Fear of vaccination						
Not at all	379 (52.2%)	158 (61.5%)	Ref.			
Rather not	152 (20.9%)	50 (19.5%)	1.27 (0.88–1.83)	0.208		
Do not care	18 (2.5%)	7 (2.7%)	1.07 (0.44–2.62)	0.879		
A little	140 (19.3%)	32 (12.5%)	1.82 (1.19–2.79)	0.005		
Very much	37 (5.1%)	10 (3.9%)	1.54 (0.75–3.18)	0.237		
A little + very much	177 (24.4%)	42 (16.3%)	1.76 (1.20–2.58)	0.004	1.39 (0.95–2.03)	0.093
Type of vaccine						
AstraZeneca	120 (16.5%)	15 (5.8%)	ref			
BioNTech/Pfizer	592 (81.4%)	236 (91.5%)	0.31 (0.18–0.55)	<0.001	0.33 (0.18–0.57)	<0.001
Other	15 (2.1%)	7 (2.7%)	0.27 (0.09–0.76)	0.009	0.27 (0.09–0.77)	0.015

APVR—adverse post-vaccination reaction, Junior—first years of study (1–3 for MS, 1 for NS)/up to 10 years, inclusive, of work in the profession; Senior—last years of study (4–6 years for MS, 2–3 years for NS)/more than 10 years of work in the profession.

**Table 7:** Associations between sex, age, chronic disease, seniority, fear of vaccination, the type of vaccine, and the strength of symptoms in adverse reactions after vaccination.

	Strong Symptoms	Slight Symptoms	Univariate Logistic Regression		Multivariate Logistic Regression	
	N (%)	N (%)	OR 95%CI	*p*	OR 95%CI	*p*
Total	331 (45.7%)	393 (54.3%)				
Sex						
Male	164 (69.2%)	73 (30.8%)	Ref			
Female	563 (75.3%)	185 (24.7%)	1.35 (0.98–1.87)	0.064		
Age						
≥27 y	161 (70.6%)	67 (29.4%)	Ref.			
19–26 y	566 (74.8%)	191 (25.2%)	1.23 (0.89–1.71)	0.212		
Chronic disease						
No	557 (72.3%)	213 (27.7%)	Ref.			
Yes	166 (78.7%)	45 (21.3%)	1.40 (0.97–2.02)	0.070		
Seniority						
Junior	246 (72.1%)	95 (27.9%)	ref			
Senior	962 (74.7%)	326 (25.3%)	0.31(0.09–1.03)	0.046		
Fear of vaccination						
Not at all	379 (52.2%)	158 (61.5%)	Ref.			
Rather not	152 (20.9%)	50 (19.5%)	1.27 (0.88–1.83)	0.208		
Do not care	18 (2.5%)	7 (2.7%)	1.07 (0.44–2.62)	0.879		
A little	140 (19.3%)	32 (12.5%)	1.82 (1.19–2.79)	0.005		
Very much	37 (5.1%)	10 (3.9%)	1.54 (0.75–3.18)	0.237		
A little + very much	177 (24.4%)	42 (16.3%)	1.76 (1.20–2.58)	0.004		
Type of vaccination						
AstraZeneca	120 (16.5%)	15 (5.8%)	Ref			
BioNTech/Pfizer	592 (81.4%)	236 (91.5%)	0.31 (0.18–0.55)	<0.001	0.15 (0.09–0.25)	<0.001
Other	15 (2.1%)	7 (2.7%)	0.27 (0.09–0.76)	0.009	0.33 (0.10–1.08)	0.068

Junior—first years of study (1–3 for MS, 1 for NS)/up to 10 years, inclusive, of work in the profession; Senior—last years of study (4–6 years for MS, 2–3 years for NS)/more than 10 years of work in the profession.

**Table 8:** Associations between sex, age, chronic disease, seniority, fear of vaccination, type of vaccination, and length of adverse reactions after vaccination.

	Long (≥4 Days)	Short (1–3 Days)	Univariate Logistic Regression		Multivariate Logistic Regression	
	N (%)	N (%)	OR 95%CI	*p*	OR 95%CI	*p*
	58 (8.0%)	669 (92.0%)				
Sex						
Male	9 (5.5%)	155 (94.5%)	Ref			
Female	49 (8.7%)	514 (91.3%)	0.61 (0.29–1.27)	0.181		
Age						
≥27 y	21 (13.0%)	140 (87.0%)	Ref.			
19–26 y	37 (6.5%)	529 (93.5%)	2.14 (1.22–3.78)	0.007	0.43 (0.22–0.81)	0.0098
Chronic disease						
No	38 (6.8%)	523 (93.2%)	Ref.			
Yes	20 (12.0%)	146 (88.0%)	1.89 (1.06–3.34)	0.028		
Seniority						
Junior	22 (8.9%)	224 (91.1%)	ref			
Senior	36 (7.5%)	445 (92.5%)	0.82 (0.47–1.43)	0.492		
Fear of vaccination						
Not at all	20 (5.3%)	359 (94.7%)	Ref.			
Rather not	18 (11.8%)	134 (88.2%)	2.41 (1.24–4.70)	0.008	2.73 (1.39–5.36)	0.004
Do not care	1 (5.6%)	17 (94.4%)	1.06 (0.13–8.34)	0.959		
A little	17 (12.1%)	123 (87.9%)	1.32 (0.89–1.96)	0.171		
Very much	2 (5.4%)	35 (94.6%)	1.03 (0.23–4.57)	0.973		
A little + very much	19 (10.7%)	158 (89.3%)	2.16 (1.12–4.16)	0.019	1.99 (1.01–3.90)	0.045
Type of vaccination						
AstraZeneca	15 (12.5%)	105 (87.5%)	Ref			
BioNTech/Pfizer	40 (6.8%)	552 (93.2%)	0.51 (0.27–0.95)	0.032	0.39 (0.21–0.75)	0.005
Other	3 (20.0%)	12 (80.0%)	1.75 (0.44–6.93)	0.420		

Junior—first years of study (1–3 for MS, 1 for NS)/up to 10 years, inclusive, of work in the profession; Senior—last years of study (4–6 years for MS, 2–3 years for NS)/more than 10 years of work in the profession.

**Table 9:** Attitudes towards obligatory annual vaccination against COVID-19 in four groups of vaccinated healthcare workers.

	D	N	MS	NS	Total	D vs. N	D vs. MS	D vs. NS	N vs. MS	N vs. NS	NS vs. MS.
Obligatory	66 (49.6%)	40 (32.3%)	231 (54.9%)	111 (29.8%)	448 (42.6%)	0.005	0.291	<0.001	<0.001	0.600	<0.001
Obligatory, if it is free	35 (26.3%)	26 (21.0%)	124 (29.5%)	127 (34.0%)	312 (29.7%)	0.315	0.486	0.101	0.063	0.006	0.165
Voluntary	32 (24.1%)	58 (46.8%)	66 (15.7%)	135 (36.2%)	291 (27.7%)	<0.001	0.028	0.011	<0.001	0.037	<0.001
Total	133 (100%)	124 (100%)	421 (100%)	373 (100%)	1051 (100%)						

D—doctors, N—nurses and midwives, MS—medical Students, NS—nursing and midwifery students.

## Data Availability

The dataset we analyzed for the current study is available from the corresponding author on request.

## Supplementary Materials

The following supporting information can be downloaded at: https://www.mdpi.com/article/10.3390/vaccines10030401/s1; Table S1: The survey questionnaire; Table S2: *p*-value for the chi-square test of independence between healthcare worker groups and the decision to vaccinate; Table S3: Determinants of vaccination against COVID-19 among vaccinated and non-vaccinated younger and older healthcare workers; Table S4: Stress, fear, or any objections prior to vaccination against COVID-19 among vaccinated and non-vaccinated younger and older healthcare workers; Table S5: Attitudes towards obligatory annual vaccination against COVID-19 among vaccinated and non-vaccinated younger and older healthcare workers; Figure S1: Flowchart for the study design.
