# Supplementary material for: Pre-Vaccination Stress, Post-Vaccination Adverse Reactions, and Attitudes towards Vaccination after Receiving the COVID-19 Vaccine among Health Care Workers
Source: Vaccines (Basel). 2022 Mar 6;10(3):401. doi: 10.3390/vaccines10030401 (PMC8952304; doi:10.3390/vaccines10030401)
Supplement: Supplementary file 1 [file vaccines-10-00401-s001.zip › vaccines-1586871-supplementary.pdf]

Table S1. The survey questionnaire

Questions	Answers
Did you take the COVID-19 vaccine?	• Yes<br>• No, but I intend to take it in the near future<br>• No, I don't want to take the COVID-19 vaccine
Did you feel any fear or had any objections prior to the vaccination?	• No, not at all<br>• No, but I was a little bit unsure<br>• I was neutral<br>• Yes, I felt a little nervous<br>• Yes, I was very nervous and anxious
What made you decide to take the COVID-19 vaccine in the end? (you can chose up to 2 answers) (skip this question if you are not vaccinated)	• My own health protection<br>• My family health protection<br>• It's the quickest way to going back to normal activities, i.e. contacting with family and friends<br>• My job and the safety of my patients<br>• Other reason, please give your own answer
What made you decide not to take the COVID-19 vaccine in the end? (you can chose up to 2 answers) (skip this question if you are vaccinated).	• I don't believe in COVID-19 and the pandemic<br>• I don't believe the vaccine is effective, it was produced too quickly<br>• I don't have time<br>• I am afraid of the side effects<br>• I don't want to take the vaccine, because I believe the vaccine wasn't tested long enough, there wasn't enough time to check and prove its effectiveness<br>• Other reason, please give your own answer
Which vaccine were you given? (skip this question if you weren't vaccinated)	• AstraZeneca<br>• BioNTech/Pfizer<br>• Moderna<br>• Johnson & Johnson<br>• Other reason, please give your own answer
Had the type of vaccine any influence on your decision to take the vaccination?	• No, it didn't matter to me what kind of vaccine I was going to be given<br>• Yes, I delayed the decision to take the vaccination waiting for the right vaccine<br>• I didn't have any choice and was vaccinated with the vaccine I wouldn't personally choose
Were there any side effects after the vaccination?	• Yes, after the first dose<br>• Yes, after the second dose<br>• Yes, after both doses<br>• No, there were no side effects
What side effects did you have after which ever dose of vaccine? (you can chose more than one answer)	• the symptoms were minimal, of no importance<br>• higher temperature/ fever<br>• pain in the muscles, bones, general poor feeling<br>• pain in the place of vaccination<br>• headache<br>• allergy<br>• give your own example
How serious were the side effects after the vaccination?	• They were minimal, of no importance<br>• They were bad enough, so I had to stay at home<br>• They were so bad, that not only I stayed at home but I also had to contact my GP

	• The side effects were so serious that I had to be taken to hospital
How long did you suffer the side effects after the vaccination?	• 1 day<br>• 2-3 days<br>• 4-7 days<br>• longer than one week
If the COVID-19 vaccination was recommended to be taken every year, should it be obligatory for the health professionals in your opinion?	• Yes, it should be obligatory<br>• Yes, it should be obligatory, if it is free of charge<br>• No, it should be voluntary
Sex	• Woman<br>• Man
Age	• in years ………………………………
Do you have any underlying chronic diseases?	• Yes<br>• No
Occupation	• Nurse/ Midwife<br>• Doctor<br>• Nursing/ Midwifery student<br>• Medicine student
Job/ study seniority	• (in years)…………………
Place of work (you can chose several)	• Hospital<br>• Outpatient clinic<br>• Specialist Clinic<br>• Hospital Emergency Ward<br>• Emergency<br>• The work outside the health service<br>• I don’t work yet, I am still a student
Did you have daily contact with COVID-19 patients during the pandemic?	• Yes, I worked in an institution which is or temporarily was turned into a COVID-19 institution<br>• No, but many COVID-19 patients were treated in my place of work<br>• No, but it often turned out later that the patient was infected with COVID-19<br>• No, I had no contact with COVID-19 patients

Table S2. ***P*-value for the chi-square test of independence between health care workers groups and the decision to vaccinate**

	**D *vs* N**	**D *vs* MS.**	**D *vs* NS**	**N *vs* MS**	**N *vs* NS**	**NS *vs* MS**
Decision to vaccinate	<0.001	0.060	0.009	<0.001	0.061	<0.001
Reasons for decision to vaccinate	0.142	0.001	0.004	0.054	0.777	0.001
Obligatory annual vaccination for professionals	0.001	0.087	<0.001	<0.001	0.018	<0.001
Fear prior to the vaccination	<0.001	<0.001	<0.001	<0.001	0.513	<0.001
Type of vaccination	0.288	0.133	<0.001	0.461	<0.001	<0.001
Influence of the type of vaccine on vaccination decisions	<0.001	0.430	<0.001	<0.001	0.238	<0.001
Occurrence of adverse reaction after vaccination	0.241	0.857	0.002	0.342	0.379	<0.001
Type of adverse reaction after vaccination	0.256	0.332	0.148	0.005	0.017	0.214
Strength of adverse reaction after vaccination	0.631	0.78	0.073	0.182	0.024	0.002
Duration of adverse reaction after vaccination	0.531	0.026	0.641	0.002	0.381	0.001

D- Doctors, N- Nurses and Midwives , MS – Medical Students, NS - Nursing and Midwifery students

Table S3. Determinants of vaccination against COVID-19 among the vaccinated and not-vaccinated younger and older health care workers.

	D N=135 (12.5%)		*p* (for Z test)	N N=128 (11.8%)		*p* (for Z test)	MS N=423 (39.2%)		p (for Z test)	NS N=394 (36.5%)		p (for Z test)	Total N=1080 (100%)		p (for Z test)
	**Junior (69)**	**Senior (66)**		**Junior (49)**	**Senior (79)**		**Junior (66)**	**Senior (357)**		**Junior (207)**	**Senior (187)**		**Junior (391)**	**Senior (689)**	
Vaccinated	66 (95.7%)	62 (93.9%)	0.654	38 (77.6%)	63 (79.7%)	0.768	63 (95.5%)	353 (98.9%)	0.046	174 (84.1%)	166 (88.8%)	0.175	341 (87.2%)	644 (93.5%)	0.001
My own protection	43 (36.4%)	51 (41.8%)	0.396	18 (26.9%)	39 (36.4%)	0.192	32 (29.1%)	201 (30.7%)	0.737	82 (29.9%)	90 (32.4%)	0.535	175 (30.8%)	381 (32.8%)	0.395
My family protection	42 (35.6%)	29 (23.8%)	0.046	20 (29.9%)	31 (29.0%)	0.902	41 (37.3%)	250 (38.2%)	0.858	77 (28.1%)	92 (33.1%)	0.204	180 (31.6%)	402 (34.6%)	0.221
Going back to normality	17 (14.4%)	14 (11.5%)	0.499	18 (26.9%)	13 (12.1%)	0.015	25 (22.7%)	118 (18.0%)	0.241	63 (23.0%)	46 (16.5%)	0.058	123 (21.6%)	191 (16.4%)	0.009
the safety of my patients	16 (13.6%)	28 (23.0%)	0.061	10 (14.9%)	22 (20.6%)	0.352*	11 (10.0%)	82 (12.5%)	0.455*	45 (16.4%)	41 (14.7%)	0.588	82 (14.4%)	173 (14.9%)	0.793
Other	0 (0.0%)	0 (0.0%)	-	1 (1.5%)	2 (1.9%)	-	1 (0.9%)	4 (0.6%)	-	7 (2.6%)	9 (3.2%)	0.633*	9 (1.6%)	15 (1.3%)	0.627*
Total	118 (100.0%)	122 (100.0%)	Ref	67 (100.0%)	107 (100.0%)	Ref	110 (100.0%)	655 (100.0%)		274 (100.0%)	278 (100.0%)	Ref	569 (100.0%)	1162 (100.0%)	Ref
Not yet vaccinated	2 (2.9%)	3 (4.5%)	0.613	7 (14.3%)	5 (6.3%)	0.136	2 (3.0%)	2 (0.6%)	0.057	12 (5.8%)	5 (2.7%)	0.128	23 (5.9%)	15 (2.2%)	0.002
I don't believe in COVID-19 and the pandemic	0 (0.0%)	0 (0.0%)	-	1 (12.5%)	0 (0.0%)	-	0 (0.0%)	0 (0.0%)	-	1 (4.3%)	0 (0.0%)	-	2 (5.7%)	0 (0.0%)	-
I don't believe the vaccine is effective	0 (0.0%)	0 (0.0%)	-	2 (25.0%)	0 (0.0%)	-	0 (0.0%)	1 (50.0%)	-	7 (30.4%)	3 (30.0%)	-	9 (25.7%)	4 (18.2%)	0.512*
I don't have time	2 (100.0%)	0 (0.0%)	-	1 (12.5%)	0 (0.0%)	-	0 (0.0%)	0 (0.0%)	-	2 (8.7%)	1 (10.0%)	-	5 (14.3%)	1 (4.5%)	-
I am afraid of the side effects	0 (0.0%)	0 (0.0%)	-	1 (12.5%)	1 (14.3%)	-	1 (50.0%)	1 (50.0%)	-	7 (30.4%)	4 (40.0%)	-	9 (25.7%)	6 (27.3%)	0.897*
Vaccine wasn't tested long enough	0 (0.0%)	0 (0.0%)	-	3 (37.5%)	2 (28.6%)	-	1 (50.0%)	0 (0.0%)	-	6 (26.1%)	2 (20.0%)	-	10 (28.6%)	4 (18.2%)	-
Other	0 (0.0%)	3 (100.0%)	-	0 (0.0%)	4 (57.1%)	-	0 (0.0%)	0 (0.0%)	-	0 (0.0%)	0 (0.0%)	-	0 (0.0%)	7 (31.8%)	-
Total	2 (100.0%)	3 (100.0%)	Ref	8 (100.0%)	7 (100.0%)	Ref	2 (100.0%)	2 (100.0%)	Ref	23 (100.0%)	10 (100.0%)	Ref	35 (100.0%)	22 (100.0%)	Ref

Not want vaccinated	1 (1.4%)	1 (1.5%)	0.975	4 (8.2%)	11 (13.9%)	0.327	1 (1.5%)	2 (0.6%)	0.396	21 (10.1%)	16 (8.6%)	0.590	27 (6.9%)	30 (4.4%)	0.072
I don’t believe in COVID-19 and the pandemic	0 (0.0%)	0 (0.0%)	-	1 (12.5%)	3 (18.8%)	-	1 (100.0%)	0 (0.0%)	-	1 (3.1%)	1 (4.2%)	-	3 (7.1%)	4 (9.3%)	0.718*
I don’t believe the vaccine is effective	0 (0.0%)	0 (0.0%)	-	3 (37.5%)	3 (18.8%)	-	0 (0.0%)	0 (0.0%)	-	7 (21.9%)	4 (16.7%)	-	10 (23.8%)	7 (16.3%)	0.388*
I don’t have time	0 (0.0%)	0 (0.0%)	-	0 (0.0%)	0 (0.0%)	-	0 (0.0%)	0 (0.0%)	-	3 (9.4%)	1 (4.2%)	-	3 (7.1%)	1 (2.3%)	-
I am afraid of the side effects	0 (0.0%)	0 (0.0%)	-	1 (12.5%)	5 (31.3%)	-	0 (0.0%)	0 (0.0%)	-	12 (37.5%)	7 (29.2%)	-	13 (31.0%)	12 (27.9%)	0.759*
Vaccine wasn’t tested long enough	1 (100.0%)	1 (100.0%)	-	3 (37.5%)	5 (31.3%)	-	0 (0.0%)	1 (50.0%)	-	7 (21.9%)	9 (37.5%)	-	11 (26.2%)	16 (37.2%)	0.278*
Other	0 (0.0%)	0 (0.0%)	-	0 (0.0%)	0 (0.0%)	-	0 (0.0%)	1 (50.0%)	-	2 (6.3%)	2 (8.3%)	-	2 (4.8%)	3 (7.0%)	-
Total	1 (100.0%)	1 (100.0%)	Ref	8 (100.0%)	16 (100.0%)	Ref	1 (100.0%)	2 (100.0%)	Ref	32 (100.0%)	24 (100.0%)	Ref	42 (100.0%)	43 (100.0%)	Ref

D- Doctors, N- Nurses and Midwives , MS – Medical Students, NS - Nursing and Midwifery students; Junior- first years of study (1-3 for MS, 1 for NS) / up to 10 years inclusive of work in the profession; Senior- last years of study (4-6 years for MS, 2-3 years for NS) / more than 10 years of work in the profession; * - low-credibility data due to the low size of the compared groups; - no data in a given group for statistical analysis

Table S4. Stress, feel of fear or any objections prior to the vaccination against COVID-19 among the vaccinated and not-vaccinated younger and older health care workers.

	D N=128 (13.0%)		p (for Z test)	N N=101 (10.3%)		p (for Z test)	SM N=416 (42.2%)		p (for Z test)	SP N=340 (34.5%)		p (for Z test)	Total N=985 (100%)		p (for Z test)
	Junior	Senior		Junior	Senior		Junior	Senior		Junior	Senior		Junior	Senior	
No, not at all	42 (63.6%)	44 (71.0%)	0.379	12 (31.6%)	30 (47.6%)	0.116*	31 (49.2%)	254 (72.0%)	<0.001	57 (32.8%)	67 (40.4%)	0.146	142 (41.6%)	395 (61.3%)	<0.001
No, I was a little bit unsure	12 (18.2%)	5 (8.1%)	0.094*	13 (34.2%)	7 (11.1%)	0.006*	17 (27.0%)	52 (14.7%)	0.016	46 (26.4%)	50 (30.1%)	0.451	88 (25.8%)	114 (17.7%)	0.003
I was neutral	6 (9.1%)	5 (8.1%)	0.836*	2 (5.3%)	0 (0.0%)	-	0 (0.0%)	3 (0.8%)	-	4 (2.3%)	5 (3.0%)	0.682*	12 (3.5%)	13 (2.0%)	0.155
Yes, I felt a little nervous	4 (6.1%)	5 (8.1%)	0.658*	9 (23.7%)	20 (31.7%)	0.388*	12 (19.0%)	38 (10.8%)	0.063*	47 (27.0%)	37 (22.3%)	0.314	72 (21.1%)	100 (15.5%)	0.028
Yes, I was very nervous and anxious	2 (3.0%)	3 (4.8%)	0.599*	2 (5.3%)	6 (9.5%)	0.444*	2 (3.2%)	5 (1.4%)	0.318*	20 (11.5%)	7 (4.2%)	0.014*	26 (7.6%)	21 (3.3%)	0.002
No answer	0 (0.0%)	0 (0.0%)	-	0 (0.0%)	0 (0.0%)	-	1 (1.6%)	1 (0.3%)	0.169*	0 (0.0%)	0 (0.0%)	-	1 (0.3%)	1 (0.2%)	0.647*
Total	66 (100.0%)	62 (100.0%)	Ref.	38 (100.0%)	63 (100.0%)	Ref.	63 (100.0%)	353 (100.0%)	Ref.	174 (100.0%)	166 (100.0%)	Ref.	341 (100.0%)	644 (100.0%)	Ref.

D- Doctors, N- Nurses and Midwives , MS – Medical Students, NS - Nursing and Midwifery students; Junior- first years of study (1-3 for MS, 1 for NS) / up to 10 years inclusive of work in the profession; Senior- last years of study (4-6 years for MS, 2-3 years for NS) / more than 10 years of work in the profession *- *low*-credibility data due to the low size of the compared groups, - no data in a given group for statistical analysis.

Table S5. Attitude towards the obligatory annual vaccination against COVID-19 among the vaccinated and not-vaccinated younger and older health care workers.

	D N=133 (12.7%)		p (for Z test)	N N=124 (11.8%)		p (for Z test)	SM N=420 (40.0%)		p (for Z test)	SP N=372 (34.5%)		p (for Z test)	Total N=1049 (100%)		p (for Z test)
	**Junior**	**Senior**		**Junior**	**Senior**		**Junior**	**Senior**		**Junior**	**Senior**		**Junior**	**Senior**	
Obligatory	36 (52.9%)	30 (46.2%)	0.435	12 (25.5%)	28 (36.4%)	0.213*	32 (50.0%)	199 (55.9%)	0.383	54 (27.8%)	57 (32.0%)	0.378	134 (35.9%)	314 (46.4%)	0.001
Obligatory, if it is free	16 (23.5%)	19 (29.2%)	0.457	15 (31.9%)	11 (14.3%)	0.021*	17 (26.6%)	106 (29.8%)	0.603	66 (34.0%)	61 (34.3%)	0.960	114 (30.6%)	197 (29.1%)	0.630
Voluntary	16 (23.5%)	16 (24.6%)	0.884	20 (42.6%)	38 (49.4%)	0.463	15 (23.4%)	51 (14.3%)	0.066	74 (38.1%)	60 (33.7%)	0.374	125 (33.5%)	165 (24.4%)	0.002
Total	68 (100.0%)	65 (100.0%)	Ref	47 (100.0%)	77 (100.0%)	Ref	64 (100.0%)	356 (100.0%)	Ref	194 (100.0%)	178 (100.0%)	Ref	373 (100.0%)	676 (100.0%)	Ref.

D- Doctors, N- Nurses and Midwives , MS – Medical Students, NS - Nursing and Midwifery students; Junior- first years of study (1-3 for MS, 1 for NS) / up to 10 years inclusive of work in the profession; Senior- last years of study (4-6 years for MS, 2-3 years for NS) / more than 10 years of work in the profession; *- low-credibility data due to the low size of the compared group

Figure S1. Flowchart for the study design

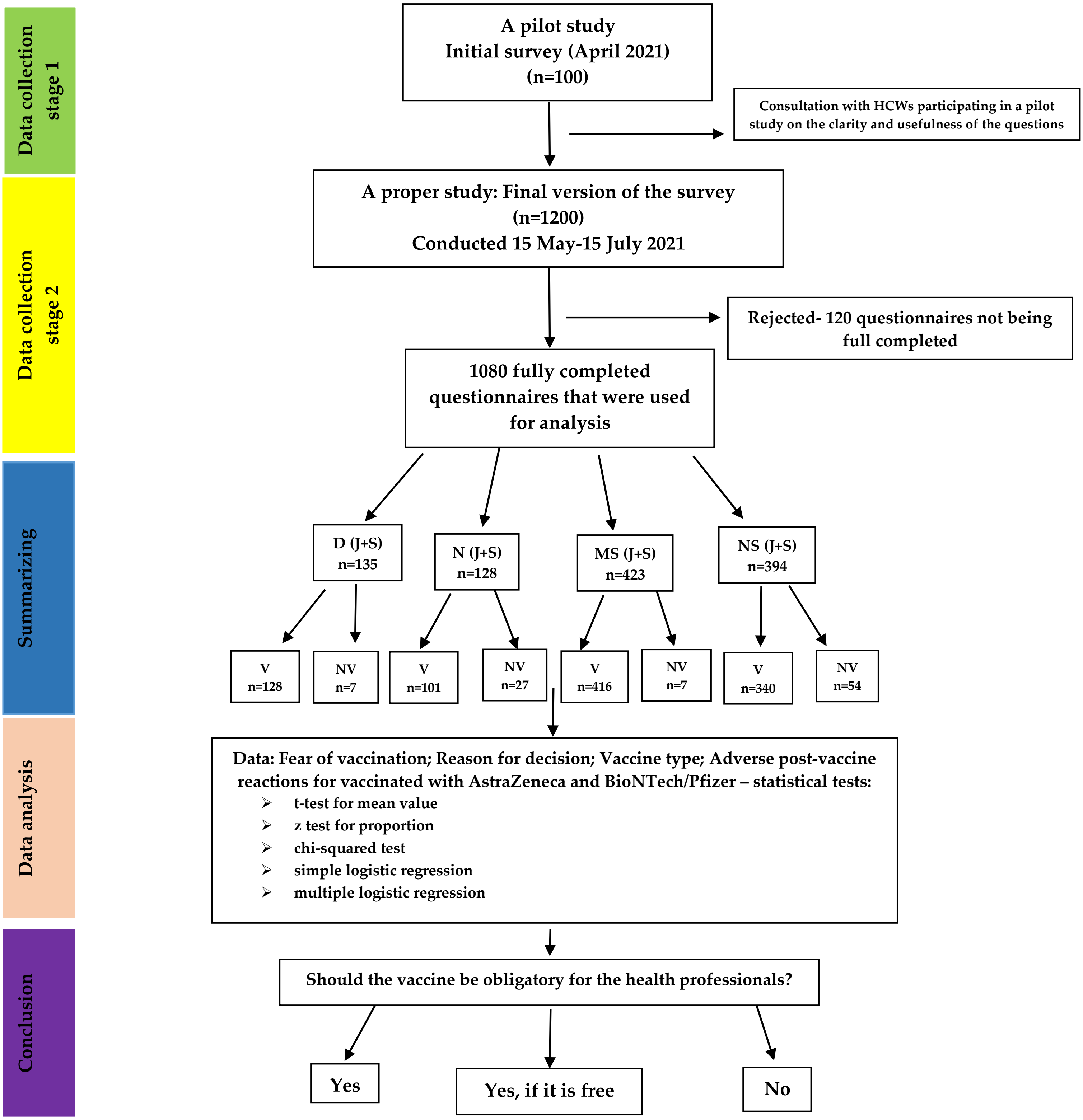

D- Doctors, N- Nurses and Midwives , MS – Medical Students, NS - Nursing and Midwifery students; Junior- first years of study (1-3 for MS, 1 for NS)/up to 10 years inclusive of work in the profession; Senior- last years of study (4-6 years for MS, 2-3 years for NS)/more than 10 years of work in the profession; V- vaccinated, NV- non-vaccinated
